# Simulated human digestion of N1-aryl-2-arylthioacetamidobenzimidazoles and their activity against Herpes-simplex virus 1 *in vitro*

**DOI:** 10.1371/journal.pone.0216384

**Published:** 2019-05-02

**Authors:** Giuseppina Mandalari, Carlo Bisignano, Antonella Smeriglio, Marcella Denaro, Maria Musarra-Pizzo, Rosamaria Pennisi, Francesca Mancuso, Stefania Ferro, Domenico Trombetta, Anna Maria Monforte, Maria Teresa Sciortino, Laura De Luca

**Affiliations:** 1 Department of Chemical, Biological, Pharmaceutical and Environmental Science, University of Messina, Messina, Italy; 2 Department of Biomedical, Dental, Morphological and Functional Images Sciences, University of Messina, Messina, Italy; Cornell University, UNITED STATES

## Abstract

Drug performance in the gastrointestinal tract (GIT) plays a crucial role in determining release and absorption. In the present work, we assessed the *in vitro* digestion of two synthetic N1-aryl-2-arylthioacetamidobenzimidazoles (NAABs), NAAB-496 and NAAB-503, using bio-relevant models of the human stomach and small intestine. The activity of NAAB-496 and NAAB-503 against herpes simplex virus (HSV-1) replication was also investigated. NAAB-496 was resistant to pepsin in the gastric environment, with a virtual 100% recovery, which decreased to 43.2% in the small intestine. NAAB-503 was sensitive to pepsin, with 65.7% degradation after 120 min gastric phase. ^1^H Nuclear magnetic resonance (NMR) post *in vitro* digestion highlighted an alteration of NAAB-496 after the gastric phase, whereas NAAB-503 appeared comparable to the original spectral data. Both NAAB-496 and NAAB-503 revealed some antiviral activity anti-HSV-1. The 50% effective concentration (EC_50_) of the compounds was 0.058 mg/mL for NAAB-496 and 0.066 for NAAB-503. Future studies will evaluate the behavior of NAAB-496 within pharmaceutical formulations.

## Introduction

The behaviour of oral drug formulations in the gastrointestinal tract (GIT) affects their disintegration, dissolution and release profiles. Standard pharmacopeial test methods and more biorelevant technologies have been applied to investigate the disintegration and dissolution of a formulation within the GIT [1–2]. Vardakou et al. [3] investigated the comparison of antral grinding forces present in the human stomach (shear forces and turbulent flow) between the USP dissolution apparatus II and a biorelevant dynamic gastric model (DGM) with human *in vivo* data. Results demonstrated that the DGM represents a close simulation of the human gastric processing forces, allowing for an imitation of the biochemical conditions and the grinding forces found in the human stomach. The DGM is a computer-controlled design able to replicate the real-time changes in pH, enzyme addition, shearing, mixing, and retention time of an adult human stomach [4].

We have previously reported the design and synthesis of a series of N_1_-aryl-benzimidazoles 2-substituted (NAABs) as inhibitors of human immunodeficiency virus type-1 (HIV-1) [5] at submicromolar and nanomolar concentration, acting as HIV-1 non-nucleoside RT inhibitors (NNRTIs). Recently, a series of novel NAABs with introduced structural modifications were tested on RT inhibition in order to increase their antiviral activity [6].

**Fig 1:**
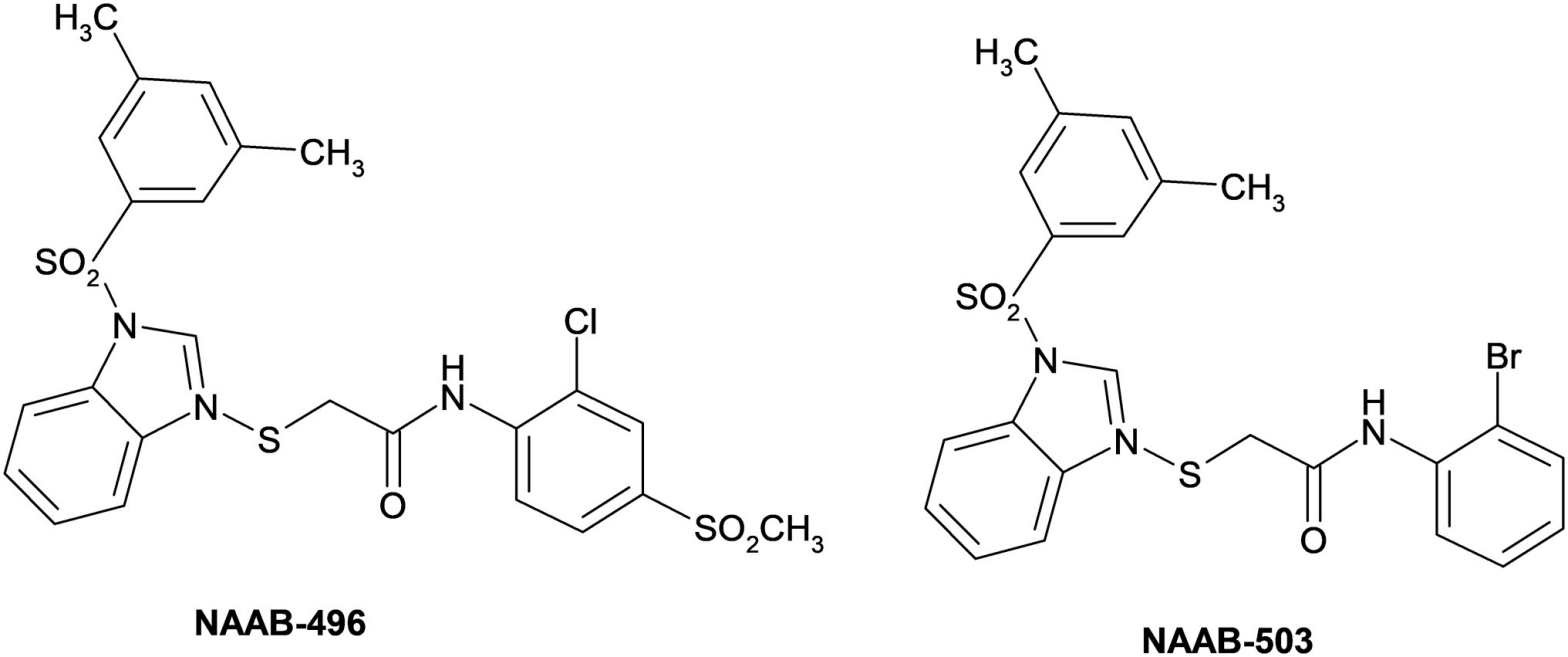
Chemical structures of compounds NAAB-496 and NAAB-503.

In the present paper, we report the simulated human digestion of the two selected NAABs (NAAB-496 and NAAB-503) depicted in Fig 1, whose activity against HIV-1NNRTIs has previously been proven [5]. A standardised protocol mimicking conditions found in the GIT was applied to investigate the stability of NAAB-496 and NAAB-503 under gastric and small intestinal conditions [7–8]. Furthermore, the antiviral potential of NAAB-496 and NAAB-503 against herpes simplex virus 1 (HSV-1) replication was evaluated. Since HSV infection is widely becoming one of the world’s most prevalent sexually-transmitted infections and drug-resistance strains frequently develop after therapeutic treatment, the discovery of novel anti-HSV drugs deserves great effort [9].

## Materials and methods

### Chemistry

Melting points were determined on a BUCHI Melting Point B-545 apparatus and corrected. Elemental analyses (C, H, N) were carried out on a Carlo Erba Model 1106 Elemental Analyzer and the results were within ±0.4% of the theoretical values and purity of tested compounds was >95%. Merck silica gel 60 F254 plates were used for TLC; column chromatography was performed on Merck silica gel 60 (230–400 mesh) and Flash Chromatography (FC) on Biotage SP1 EXP. Compounds NAAB-496 and NAAB-503 were prepared following a synthetic procedure previously reported by us and spectral data are in accordance with the literature [5].

In addition, we report 13C-NMR data of NAAB-496 and NAAB-503 derivatives. 2-({1-[(3,5-Dimethylphenyl)sulfonyl]-1H-benzimidazol-2-yl]sulfanyl}-N-[2-chloro-4 (methylsulfonyl)phenyl]-Acetamide (NAAB-496): 13C-NMR (CDCl3) (δ): 166.3 (CO), 141.5, 140.1, 139.6, 137.2, 136.3, 135.9, 135.5, 131.5, 128.9, 128.6, 127.8, 126.6, 126.2, 124.0, 124.4, 123.3, 115.4, 112.7, 47.7, 36.3, 20.6.

### Simulated human digestion

*In vitro* gastric and duodenal digestion of NAAB-496 and NAAB-503 was performed as previously described [7–8]. Briefly, NAAB-496 and NAAB-503 were dissolved in simulated gastric acid solution containing HCl (0.2 M), NaCl (0.08 M), CaCl_2_ (0.03 mM), and NaH_2_PO_4_ (0.9 mM) at the concentration of 1 mg/mL and the pH was adjusted to 2.5. A solution of single shelled lecithin liposomes prepared as previously described [7], porcine gastric mucosa pepsin and a gastric lipase analogue from *Rhizopus oryzae* were added at the final concentration of 0.127 mM, 9,000 U/mL and 60 U/mL, respectively. Duodenal digestions were performed at pH 6.8 with addition of a simulated bile solution containing lecithin (6.5 mM), cholesterol (4 mM), sodium taurocholate (12.5 mM), and sodium glycodeoxycholate (12.5 mM) in a salt solution made of NaCl (146.0 mM), CaCl_2_ (2.6 mM), and KCl (4.8 mM) and simulated pancreatic juice containing NaCl (125.0 mM), CaCl_2_ (0.6 mM), MgCl_2_ (0.3 mM), and ZnSO_4_ • 7H_2_O (4.1 μM). Porcine pancreatic lipase, porcine colipase, porcine trypsin, bovine α-chymotrypsin and porcine α-amylase were added to the pancreatic juice so that the final enzyme concentrations were the following: pancreatic lipase (590 U/mL), porcine colipase (3.2 μg/mL), porcine trypsin (11 U/mL), bovine α-chymotrypsin (24 U/mL) and porcine α-amylase (300 U/mL).

Aliquots (100 μl) were withdrawn at several time-points during gastric (0.1, 1, 2, 5, 10, 20, 40, 60, 90 and 120 min) and duodenal (0.1, 1, 2, 5, 10, 15, 30 and 60 min) digestion.

### Sample preparation

All time-point samples were thawed at room temperature (RT) and extracted with HPLC grade acetonitrile (1:1 v/v) mixing for 3 min in order to solubilize the molecules of interest and, at the same time, to precipitate the proteins thus avoiding any interference. Samples were centrifuged at 12,000 *g* for 5 min at 4°C (Heraeus Biofuge Primo R Centrifuge, Thermo Scientific, Italy), each supernatant was then filtered through a 0.22μm filter membrane and injected into the HPLC-DAD system.

### HPLC-DAD analysis

Time-point sample extracts were analysed using a Varian Res-Elut C18 reverse phase column (150 x 4.6 mm, 5μm; Varian, Harbor City, CA, USA) by an Agilent HP1100 system (Agilent Ltd, West Lothian, UK) coupled with a photodiode array detector and a binary pump. An isocratic elution using two solvents—solvent A) Water and solvent B) Acetonitrile, both HPLC grade, was applied in order to analyse the two molecules as follows: NAAB-496 (A = 30%; B = 70%) and NAAB-503 (A = 20%; B = 80%). The flow rate was 1 mL/min, and the thermostatically controlled autosampler and column oven were set at 10°C and 25°C, respectively. The detection conditions were set at 280 nm. The UV-Vis spectra were recorded from 200 to 400 nm. Data acquisition was performed using ChemStation A.10.01 software (Agilent, USA). The identification was made according to UV-visible spectra, retention time and co-elution with reference standards. Quantification was carried out by external standard calibration curves (0.039–0.625 μg/mL) and expressed as average ± SD of three independent experiments (n = 3).

### Method validation

**Fig 2:**
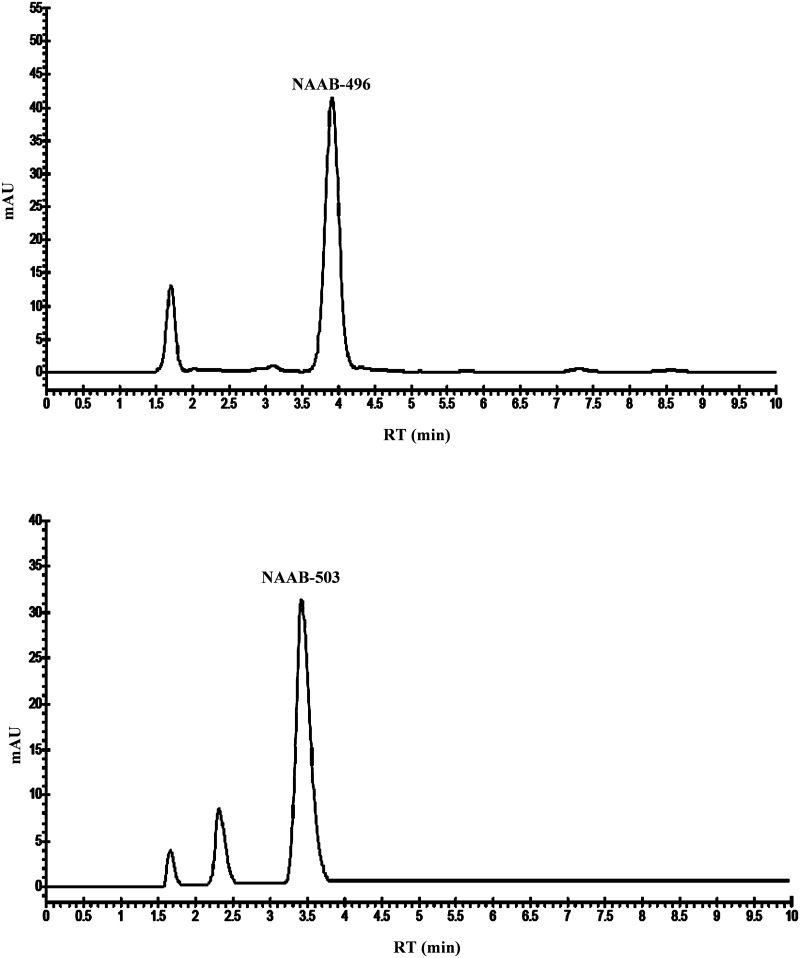
Representative chromatograms of NAAB-496 and NAAB-503 reference standard solutions (10 μg/mL).

**Fig 3:**
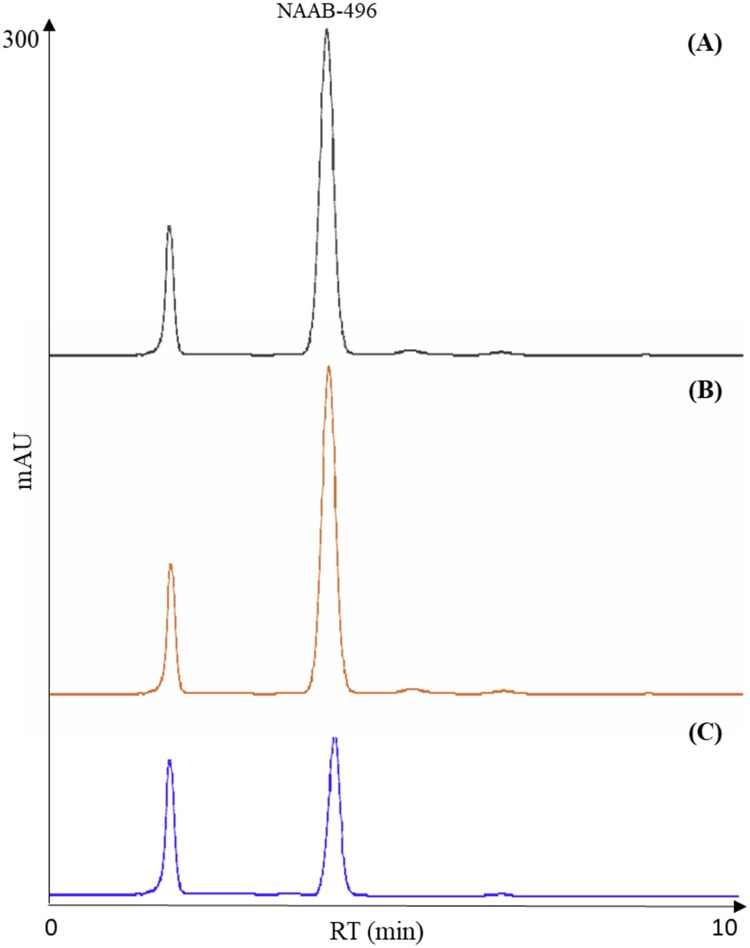
Representative chromatograms of NAAB-496 (A) degradation after gastric (B) and gastric plus duodenal digestion (C).

**Fig 4:**
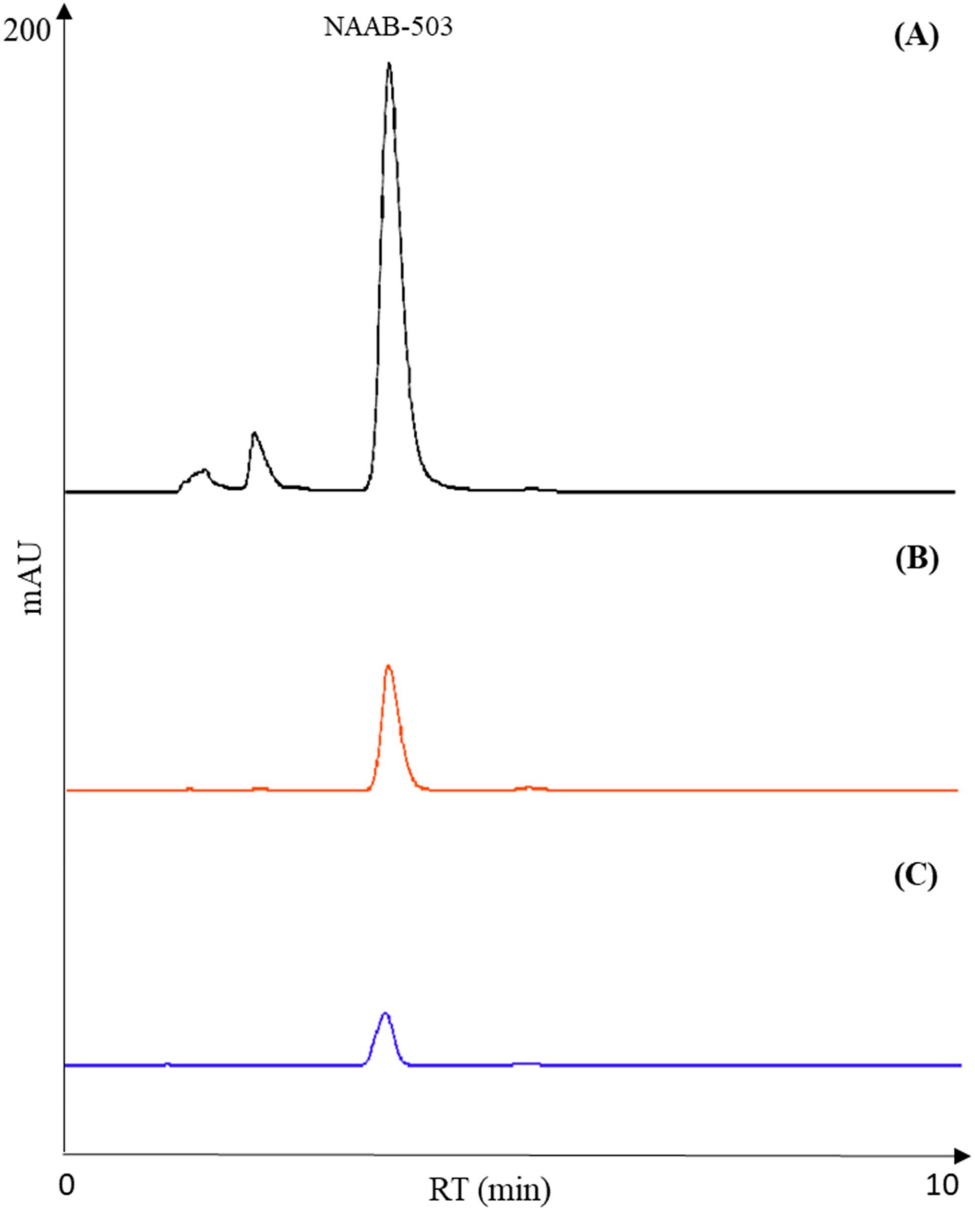
Representative chromatograms of NAAB-503 (A) degradation after gastric (B) and gastric plus duodenal digestion (C).

HPLC methods were validated according to ICH Harmonised Tripartite Guidelines [10], in terms of selectivity, linearity, limit of detection, quantitation and precision. A good analytical method should be able to accurately measure the analytes in the presence of suspected interferences such as its own degradation products and any co-eluting compounds. The chromatographic separation of NAAB-496 and NAAB-503 did not show any overlap (base-line separations) (Fig 2). Furthermore, as highlighted in the chromatogram, no interferences from matrix constituents were found at the retention times of chemicals (Figs 3 and 4). A test solution with different concentrations of reference standards was prepared and analysed by using the analytical parameters described earlier. The detection limit was calculated as the amount of chemicals that resulted in a peak three times higher with respect to the baseline noise. The calibration range, linearity, detection and quantification limit and precision, expressed as twice the standard deviation of compounds of interest were calculated and the results were listed in Table 1.

**Table 1:** Method validation parameters of NAAB-503 and NAAB-496.

Molecules	LOD (μg/ml)	LOQ (μg/ml)	Precision (2*SD)	Range (μg/ml)	Linearity (R^2^)
NAAB-503	0.025	0.076	0.007	0.039–0.625	0.9998
NAAB-496	0.019	0.058	0.032	0.039–0.625	0.9998

LOD = Limit of detection

LOQ = Limit of quantification

SD = Standard deviation

### NMR analysis

^1^H NMR spectra were measured with a Varian Gemini-300 spectrometer in CDCl_3_ with TMS as internal standard or in DMSO-d_6_ and were performed on the starting material and post *in vitro* gastric and duodenal digestion.

### Cells culture and virus

VERO cell lines were cultured in minimal essential medium (EMEM), supplemented with 6% fetal bovine serum (FBS) (Lonza, Belgium) at 37°C in a CO2 incubator. HSV-1 (F) is the prototype strain kindly provided by Professor Bernard Roizman (University of Chicago). Viral stocks were propagated in Vero cells and stored at -80 °C.

### Cell proliferation assay

The cell viability of Vero cells treated with NAABs was evaluated by using ViaLight plus cell proliferation and cytotoxicity bioassay kit (Lonza Group Ltd., Basel, Switzerland). Vero cells were grown in 96-well plates and treated with different concentrations of NAABs: 0.01, 0.05, 0.06, 0.08, 0.1, 0.15, 0.2, 0.4, 0.6, 0.8 and 1.6 mg/mL. After the incubation period, the emitted light intensity related to ATP degradation was quantified with the GloMax Multi Microplate Luminometer (Promega Corporation, 2800 Woods Hollow Road Madison, WI, USA). The cell proliferation index (%) was determinate by evaluating the luminescence value of emitted light intensity related to ATP degradation according to the manufacturer’s instructions as previously described [11]. The 50% cytotoxic concentration (CC_50_) was determined by using non-linear regression analysis of concentration-effect curves by the GraphPad Prism 6 software. The data represent the means ± standard deviation of three independent experiments.

### Plaque reduction assay

The antiviral activity was evaluated by plaque reduction assay. For experimental procedure HSV-1 was diluted in Dulbecco’s Modified Eagle’s Medium (DMEM) to yield 100–90 plaques/100μL. Vero cells were grown in 6-well plate and infected with the viral inoculum for 1h at 37 °C with gently shaking. Viral inoculum was then removed and replaced with fresh culture medium containing 0.8% methylcellulose in the presence of NAABs. After 3 days, the cells were stained with crystal violet for plaque detection. The data are representative as the means of triplicates ± SD for each dilution. Acyclovir (Acv) was used as a positive control. The following final drug concentrations were used in the assay NAABs 0.01, 0.05, 0.06, 0.08, and 0.1 mg/ml and Acv 0.5, 1, 10, and 20 μM. After 3 days, the cells were stained with crystal violet for plaque detection. The plaques were counted microscopically at low power. The mean number of plaques in each well was calculated and the 50% effective concentration (EC_50_) of NAABs was determined by using non-linear regression analysis of concentration-effect curves by the GraphPad Prism 6 software. The data represent the means ± standard deviation of three independent experiments. In each case, the selectivity index (SI) was calculated from the ratio CC_50_/EC_50_.

### DNA extraction and quantitative real-time RT-PCR

Vero cells were infected with HSV-1 (F) at MOI 1 for 1 hour at 37°C. After incubation the inoculum was replaced by fresh growth medium with either NAAB-496 or NAAB-503 (0.06 mg/mL), separately. Samples were collected at 24h post infection, suspended in of TRIzol Reagent (Invitrogen) and used for DNA extraction, according to the manufacturer’s instructions. The DNA was precipitated and quantification was carried out as previously described [12]. Briefly, Real Time PCR was carried out in 25μL reaction mixture containing the forward and reverse primer (For-59-CATCACCGACCCGGAGAGGGAC; Rrev-59-GGGCCAGGCGCTTGTTGGTGTA), and 300 nM of TaqMan probe (59-6FAM100 CCGCCGAACTGAGCAGACACCCGCGC-TAMRA) in Maxima Probe qPCR Master Mix (Maxima Probe qPCR, Fermentas Life Sciences). The amplification was carried out with the aid of Cepheid SmartCycler II System (Cepheid Europe, France). Each amplification run contained two negative controls and 10-fold serially diluted reference DNA obtained from BAC-HSV in order to generate the standard curve. Viral load was derived from Ct using the standard curve generated in parallel and expressed as concentration of ng of DNA for μl.

### Statistical analysis

Student`s t-test was used for statistical analysis to compare different conditions. Results are the mean ± SD of three independent experiments (* p < 0.05, ** p < 0.01; *** p < 0.001). For the data analysis, the Graphpad Prism 6 software (GraphPad Software, San Diego, CA, USA) was used.

## Results

### HPLC-DAD analysis

Results showed that a simple and fast liquid-liquid extraction allowed isolating the compounds of interest. Furthermore, by using acetonitrile as a solvent, it was possible at the same time to obtain the precipitation of the protein fraction of the sample, avoiding any interference. As observed in Figs 3 and 4, the chromatographic profile of the samples were very close to that of the reference standard NAAB-496 and NAAB-503 (Fig 2), which were also solubilized in acetonitrile. Moreover, the two analytes (NAAB-496 and NAAB-503) appeared to be well separated and easily identifiable despite the complex matrix (Figs 3 and 4).

In conclusion, the extractive and analytical methods were fast, simple to perform and reproducible as well as selective and precise for the molecules of interest with excellent good limits of detection and quantification (Table 1).

### Kinetics of digestion of NAAB-496 and NAAB-503

**Fig 5:**
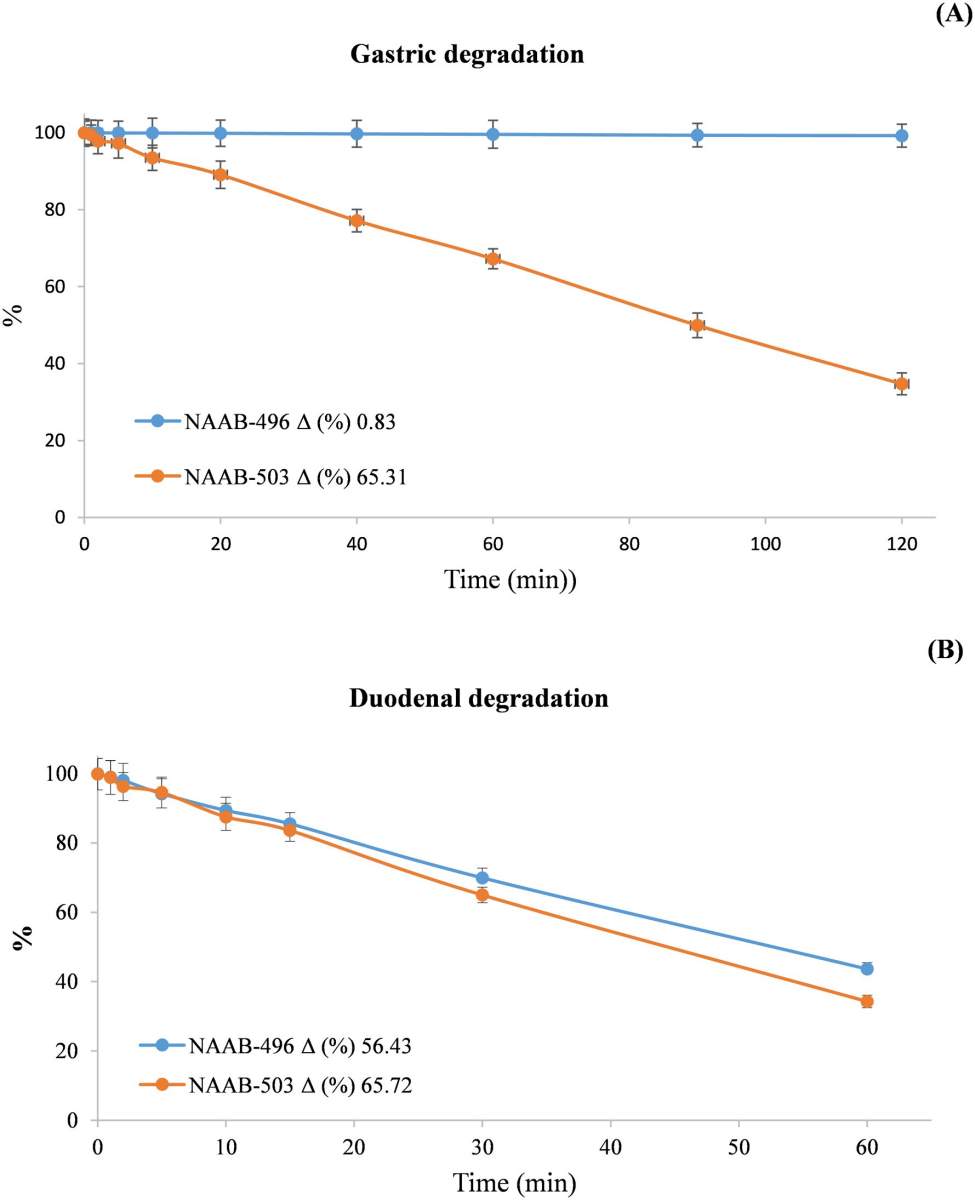
Kinetic of degradation of NAAB-496 and NAAB-503 after gastric (A) and gastric plus duodenal digestion (B).

Results presented in Fig 5A indicated a rapid degradation of NAAB-503 in the gastric environment, while NAAB–496 was pepsin resistant. While the recovery of NAAB-496 was virtually 100% under gastric digestion, recovery of NAAB-503 decreased to 89.08% after 20 min incubation, with a final 65.7% degradation after 120 min. Both molecules were resistant at pH 2.5 for 3 h, with no indication of degradation (results not shown).

Only a slight increase in NAAB-503 degradation over that observed in the gastric environment was obtained post *in vitro* gastric plus duodenal digestion (Fig 5B), demonstrating a stability of the compound to pancreatic juice enzymes. A higher duodenal solubilisation was recorded for NAAB-496, with a total degradation of 56.8% (Fig 5B).

### Nuclear magnetic resonance post *in vitro* gastric and duodenal digestion

^1^H Nuclear magnetic resonance (NMR) was applied in order to monitor the behaviour of the studied molecules NAAB-496 and NAAB-503. Specifically, ^1^H NMR spectra were performed on the residues obtained post *in vitro* gastric and duodenal digestion ant the results compared with the spectral data of the starting material.

For derivative NAAB-496 ^1^H NMR analysis highlighted an alteration of the molecule visible already post *in vitro* gastric digestion, characterized by an increase in the level of signals complexity, probably due to the fragmentation of the compound which however remained quantitatively similar to the starting material. In addition, the same trend was observed post *in vitro* duodenal digestion.

On the contrary, signals observed post *in vitro* gastric and duodenal digestion of derivative NAAB-503 appear perfectly comparable with those of original spectral data.

The obtained information suggests that the molecule remained unchanged until the end of the digestive process, although it could hypothesize a quantitative decrease.

### Cellular proliferation index and inhibition of HSV replication

**Fig 6:**
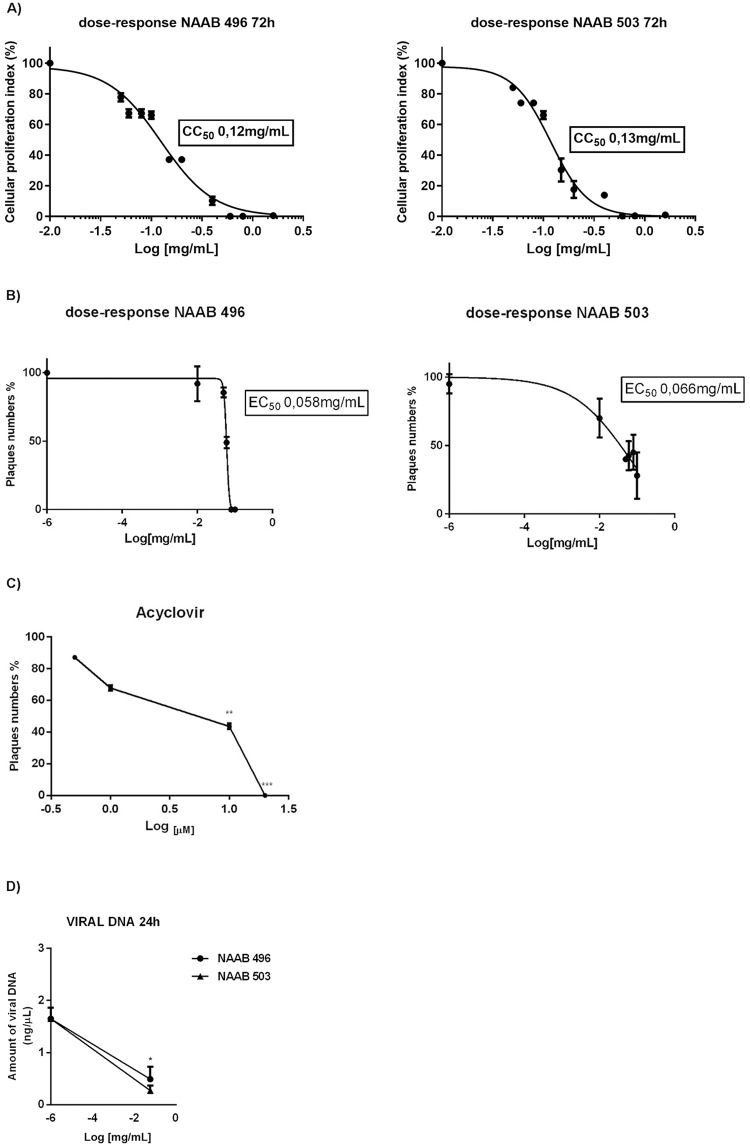
Viability assay and Antiviral activity of NAAB-496 and NAAB-503 in Vero cells. (A) The cytotoxicity effect of NAABs was evaluated on Vero cells in presence of different concentrations of NAABs compound (0.01, 0.05, 0.06, 0.08, 0.1, 0.15, 0.2, 0.4, 0.6, 0.8 and 1.6 mg/mL) separately. The cell proliferation index (%) was determined at 72 hours of treatment on the basis of ATP levels using the ViaLight Plus Cell Proliferation and Cytotoxicity BioAssay Kit (Lonza Group Ltd., Basel, Switzerland). The CC_50_ was obtained from nonlinear regression analysis of concentration-effect curves by the GraphPad Prism 6 Demo program and expressed the means ± standard deviation of three independent experiments. (B) Antiviral activity of NAAB-496 and NAAB-503 was evaluated on the basis of plaques reduction assay in Vero cells infected with HSV-1 (F) and incubated in the presence of NAAB 496 and NAAB 503 at different concentrations (0.01, 0.05, 0.06, 0.08 and 0.1 mg/mL). The EC_50_ was obtained from nonlinear regression analysis of concentration-effect curves by the GraphPad Prism 6 program. (C) Antiviral activity of Acyclovir: Vero cells infected with HSV-1 (F) and incubated for 1 h at 37 °C. After the incubation time, the inoculum was removed, and the monolayers were overlaid with DMEM containing 0.8% methylcellulose in the presence of Acyclovir at 0.5, 1, 10, and 20 μM. The cells were stained with crystal violet for plaques detection. (D) The viral DNA was extracted from Vero cells 24h post HSV-1 infection and NAABs treatment (0.06mg/mL), as described in the Materials and Methods. Quantization of viral DNA was performed using real-time quantitative PCR and using the standard curve generated in parallel and expressed as a concentration of ng/μl. Results are the mean ± SD of three independent experiments (* p < 0.05, ** p < 0.01; *** p < 0.001).

To examine the effect of NAABs on the cell system utilized, Vero cells were incubated separately with NAAB-496 and NAAB-503 in the presence of different concentrations (0.01, 0.05, 0.06, 0.08, 0.1, 0.15, 0.2, 0.4, 0.6, 0.8 and 1.6 mg/mL). Samples were collected at 72h of treatment and then the quantification of the emitted light intensity, related to ATP degradation as a cellular proliferation index, was measured. The results showed that treatment with NAAB-496 and NAAB-503 displayed a reduction on cell proliferation index at 72h of treatment at concentration ranging from 0.15 up to 1.6 mg/mL (Fig 6A).

To further explore whether NAABs can interfere with viral replication, the anti-HSV-1 activity was tested by plaque reduction assay. The experiments were performed in DMSO as a solvent and non-cytotoxic concentrations of compounds (0.01, 0.05, 0.06, 0.08, and 0.1 mg/mL) were used. The results are given in Fig 6B. Both NAAB-496 and NAAB-503 revealed an increased anti-HSV-1 activity depending on increased concentration used. The highest concentration of both compounds (ranging from 0.15 to 1.6 mg/mL) were not used because of their cytotoxic effects. The selectivity indexes (SI) of the NAAB-496 and NAAB-503 were determined by cellular proliferation index assay by calculating the ratio CC_50_ over the EC_50_. The 50% cytotoxic concentration (CC_50_) was defined as the compounds concentration that reduced the cell viability by 50% when compared to untreated controls. The 50% inhibitor concentration (EC_50_) was defined as the concentration of the compounds that inhibit 50% of plaque formation of HSV-1 when compared to the virus control. The CC_50_ an EC_50_ were obtained from nonlinear regression analysis of concentration-effect curves by the GraphPad Prism 6 program and expressed the means ± standard deviation of three independent experiments. The CC_50_ for NAAB-496 at 72h was 0,12 mg/mL while for NAAB-503 at 72h was 0,13 mg/mL. The 72h time point was referred as the incubation period for HSV plaque assay. The EC_50_ for NAAB-496 calculated at 72h was 0.058 mg/mL while for NAAB-503 calculated at 72h was 0.066 mg/mL (Table 2). The SI values of each compounds on HSV-1 were reported in Table 2. The use of acyclovir as positive control is displayed in Fig 6C. Data obtained by plaque reduction assay were confirmed by the quantification of viral DNA using RealTime PCR. The results showed that both NAAB-496 and NAAB-503 at the concentration of 0.06 mg/mL (EC_50_), were able to block viral DNA accumulation, when compared to the untreated infected cells (*p < 0.05) (Fig 6D).

**Table 2:** Selectivity index (SI) and cytotoxic and antiviral activity of NAAB-496 and NAAB-503 against HSV-1.

Molecule	CC_50 (mg/mL)_ ^a^ 72h	EC_50 (mg/mL)_ ^b^ 72h	SI^c^ 72h
NAAB 496	0.12	0.058	2.07
NAAB 503	0.13	0.066	1.97

^a^50% cytotoxic concentration;

^b^ 50% inhibitory concentration;

^c^ ratio of CC_50_ to EC_50_.

## Discussion

The digestibility of two NAABs, NAAB-496 and NAAB-503, was assessed *in vitro* under conditions found in the human stomach and small intestine. Although the structural conformation of the two molecules appears similar, NAAB-496 was generally resistant to pepsinolysis, although ^1^H NMR highlighted an alteration post gastric digestion. On the contrary, NAAB-503 showed a different pattern of digestion kinetics, being susceptible to pepsin without changes in its original spectrum. Unlike other peptidases, pepsin hydrolyses only peptide bonds, not amide or ester linkages [13]. The cleavage specificity includes peptides with an aromatic acid on either side of the peptide bond, with increased susceptibility if there is a sulfur-containing amino acid close to the peptide bond, which has an aromatic amino acid [14]. We believe the sulfonyl group in NAAB-496 may have been preferentially hydrolysed by pepsin, thus determining a change from its original spectral data. The cleavage of this bond in a protein substrate could have determined an equilibrium interaction with the enzyme with acquired resistance. The significant change in rate of digestion of NAAB-503 under physiological gastric conditions could be due to the cleavage of a different bond which did not result in an alteration of the original spectral data. No significant changes were reported in the duodenal compartment, indicating no selectivity for the two molecules by trypsin and chymotrypsin.

When evaluating the potential performance of pharmaceutical products in humans, it is important to investigate their interactions with food components, especially lipids, which may affect the rate and extent of fragments generated during digestion. Vardakou et al. [1] rupture times of a range of capsule types and gastric emptying profiles using the dynamic gastric model were longer in the fed state compared with the fasted state. Furthermore, gastric content and residence time are crucial factors determining capsule disintegration and rate of delivery in the small intestine. Curatolo et al. [15] reported an increased degradation of azithromycin in the fed state, possibly due to interaction between the capsule shell and the food present in the stomach. NAAB-496 proved to be gastric resistance; nevertheless, its behaviour will need to be evaluated within a pharmaceutical formulation.

Standard treatment of HSV infections are based on nucleoside analogues targeting viral DNA polymerase. The search for novel antiviral drugs against herpes continues because there is a more serious problem in immunocompromised patients, including HIV patients. Indeed, Herpes simplex virus type 2 (HSV-2), the causative pathogen of genital herpes, is closely associated with the occurrence of human immunodeficiency virus (HIV) infection [16]. Antiviral Effects of ABMA against Herpes Simplex Virus Type 2 in Vitro and in Vivo).

Here, we reported that NAAB-496 and NAAB-503 acting as NNRTIs [5], exert an antiviral activity against herpes simplex virus. Nevertheless, both compounds were not able to completely inhibit the virus replication. This may depend on many factors, including the bioavailability of compounds in the cellular system or the failure to reach the viral proteins target.

Based on literature data, numerous drugs inhibiting HIV displayed anti-HSV activity. Indeed, herpes simplex virus (HSV) encodes two proteins with potential RNase H-like folds, the infected cell protein 8 (ICP8) DNA-binding protein, which is necessary for viral DNA replication and the viral terminase, which is essential for viral DNA cleavage and packaging [17].

## Abbreviations

DAD: Diode Array Detector
DD: in vitro gastric plus duodenal digestion
GD: in vitro gastric digestion
GIT: gastrointestinal tract
HIV-1: human immunodeficiency virus type-1
HPLC: High Performance Liquid Chromatography
HSV-1: Herpes simplex virus 1
LOD: Limit of detection
LOQ: Limit of Quantification
NAAB: N1-aryl-2-arylthioacetamidobenzimidazoles
NNRTIs: non-nucleoside RT inhibitors
SD: Standard Deviation
